# Sex-specific diagnostic trajectories and time to transition from non-SMI to severe mental illness in Chinese adolescent inpatients

**DOI:** 10.3389/fpsyt.2026.1793421

**Published:** 2026-03-17

**Authors:** Xiang Kong, Mingjian Cai, Wenjuan Liu, You Xu, Ning Ren, Hongjing Mao

**Affiliations:** Affiliated Mental Health Center & Hangzhou Seventh People's Hospital, Zhejiang University School of Medicine, Hangzhou, China

**Keywords:** adolescents, bipolar disorder, diagnostic stability, longitudinal study, schizophrenia spectrum disorders, severe mental illness, sex differences

## Abstract

**Objective:**

Adolescence is a high-risk period for mental disorders, and early clinical presentations often show uncertainty and pluripotentiality. Longitudinal evidence on transitions from non-specific diagnoses to severe mental illness (SMI—defined as schizophrenia spectrum and bipolar disorders) in non-Western populations remains limited. Using real-world data, we aimed to characterize diagnostic stability, transitions from non-SMI to SMI, and sex- and age-related predictors in Chinese adolescent inpatients.

**Methods:**

This retrospective longitudinal cohort study utilized electronic medical records from a large tertiary psychiatric hospital in Eastern China (2010–2026). We included 884 first-time inpatients aged 12–17 years with ≥3 years of follow-up and at least two complete inpatient records. ICD-10 diagnoses were grouped into SMI and non-SMI categories. Sankey diagrams and transition matrices were used to describe diagnostic trajectories from baseline to the last admission. Among patients with non-SMI at baseline, Kaplan–Meier analyses examined the time to transition to SMI, and multivariable logistic regression tested the independent effects of sex and baseline age on SMI conversion.

**Results:**

Over a median follow-up of 4.60 years (IQR 3.63–6.53), SMI diagnoses showed high stability: 81%(243/300)of schizophrenia spectrum disorders and 74%(104/141) of bipolar disorders remained unchanged. Overall, 39.2%(346/884)of patients experienced at least one diagnostic change, primarily within non-SMI categories; depressive disorders were the most frequent antecedent of bipolar disorder (13%(22/171) converted). Sex-stratified analyses suggested that certain externalising and obsessive–compulsive presentations in males, and internalising and stress-related presentations in females, were more frequently followed by SMI; however, several subgroup estimates were based on small numbers and should be considered exploratory. Kaplan–Meier curves indicated that the risk of transition from non-SMI to SMI clustered between 4 and 8 years after the first admission. Each 1-year increase in baseline age was associated with a 38% higher risk of SMI conversion (*OR* = 1.38, 95% *CI* 1.19–1.60, *P* < 0.001), and, after adjusting for age, males had approximately twice the risk of SMI conversion compared with females (*OR* = 1.90, 95% *CI* 1.17–3.07, *P* = 0.009).

**Conclusions:**

Adolescent psychiatric diagnoses show substantial longitudinal evolution, with relatively stable SMI once established but appreciable medium- to long-term progression from non-SMI to SMI. The identified sex-specific pathways and the 4-to-8-year high-risk window support longitudinal, developmentally informed monitoring—particularly for older male adolescents with severe or atypical non-SMI presentations.

## 1 Introduction

Adolescence is a critical period for the onset of mental disorders. Many severe mental illnesses (SMI), particularly schizophrenia and bipolar disorder, emerge during this stage or are preceded by prodromal symptoms in childhood and adolescence (1–3). A large meta-analysis of 192 epidemiological studies showed that the age at onset of most mental disorders is concentrated in childhood and adolescence, with psychotic and affective psychotic disorders typically peaking in late adolescence or early adulthood (1). Early identification and intervention within this developmental window can improve long-term outcomes (3, 4). However, in real-world child and adolescent mental health services (CAMHS), early clinical presentations are often broad, overlapping, and atypical, making it difficult to establish accurate and stable diagnoses at a single time point (5, 6).

Longitudinal follow-up studies consistently demonstrate substantial diagnostic instability in child- and adolescent-onset psychiatric disorders. While diagnoses of schizophrenia and bipolar disorder in adulthood are generally relatively stable over the medium to long term (6–9), initial diagnoses in early-onset cases are frequently revised over several years of follow-up (4, 7–9). For example, a two-year follow-up of early-onset first-episode psychosis found that around one-third of patients underwent a diagnostic change (9), and a five-year follow-up of adolescent inpatients showed that psychotic and autism spectrum disorders were relatively stable, whereas depressive, anxiety, and behavioural disorders were more likely to convert (4). These observations have been described using terms such as diagnostic heterogeneity, clinical fluidity, and pluripotentiality, but they converge on a common clinical challenge: many child and adolescent clinical syndromes represent shared antecedent states that can evolve into different SMI outcomes over time (3, 10–12).

The diagnostic conversion from depressive disorder to bipolar disorder is a frequently cited example of such longitudinal evolution. Clinical and epidemiological studies have shown that a subset of adolescents with major depressive disorder later develop bipolar disorder during follow-up (8, 13–15), with the risk influenced by age, sex, and psychiatric comorbidities (13). Delayed revision from a unipolar to a bipolar diagnosis may lead to inappropriate treatment and worse outcomes (8, 13–15). Similarly, diagnoses within the early-onset psychotic spectrum are not always stable, with some cases converting over time between schizophrenia, schizoaffective disorder, and affective psychosis (6, 9, 16). Systematic research on the stability of child and adolescent diagnoses, their conversion patterns, and associated predictors is therefore crucial for refining early diagnosis and implementing staged interventions.

In clinical and public-health practice, schizophrenia spectrum and bipolar disorders are often regarded as prototypical severe mental illnesses because of their typical onset in adolescence or early adulthood, high relapse risk, chronic or recurrent course, and substantial long-term functional and societal burden. By contrast, the wide range of non-SMI diagnoses commonly seen in adolescent inpatient services (e.g. depressive, anxiety, obsessive–compulsive, externalising, and stress-related disorders) can be conceptualised as diverse, often non-specific presentations from which these prototypical SMIs may emerge.

CAMHS, particularly inpatient services, provide a risk-enriched context in which to study diagnostic stability and evolution. Adolescent inpatients tend to have more severe symptoms, higher comorbidity, and greater functional impairment, and are at increased risk of developing psychotic or bipolar disorders during follow-up (4, 5, 17). In a nationwide Finnish birth cohort, receipt of CAMHS—especially inpatient care or emergency psychiatric assessment—was associated with a substantially increased subsequent risk of psychotic or bipolar disorders, suggesting that early service contact itself may serve as a marker for youth at high risk for SMI (17).

Despite a growing international literature on diagnostic stability among adolescent inpatients and early-onset psychosis (4, 7, 9), several gaps remain. Most existing studies are from high-income Western countries, and there is little evidence from East Asia, particularly China, where service systems and diagnostic practices may differ (1, 5). Many studies have focused on a single diagnostic group (e.g. early-onset psychosis or paediatric bipolar disorder) rather than adopting a broader spectrum perspective to compare the stability of SMI versus non-SMI diagnoses and to quantify cross-spectrum conversion pathways. Evidence is also limited and inconsistent regarding which baseline characteristics—such as sex, age at first diagnosis, and initial diagnostic spectrum—predict medium- to long-term conversion to SMI and the timing of such transitions (4, 11–15, 17).

To address these gaps, we constructed a retrospective longitudinal cohort of adolescent inpatients aged 12–17 years, using 16 years of electronic medical record data from a large tertiary psychiatric hospital in eastern China. Within the confines of a single inpatient system, the primary objectives of this study were: (1) to describe diagnostic trajectories from first admission through long-term follow-up, and to compare the stability of SMI versus non-SMI inpatient diagnoses; (2) to quantify specific conversion pathways and proportions from non-SMI diagnoses to SMI (schizophrenia spectrum and bipolar disorder), and to identify the main upstream inpatient diagnostic categories that precede SMI; and (3) to examine, using survival analyses and multivariable regression models, the roles of sex, age at first inpatient diagnosis, and initial diagnostic spectrum in predicting SMI conversion risk and its timing. Our goal is to provide real-world evidence on inpatient diagnostic stability and evolution in Chinese adolescents and to inform developmentally and gender-sensitive strategies for early identification, risk stratification, and follow-up.

## 2 Methods

### 2.1 Study design and data source

This was a retrospective, longitudinal cohort study based on real-world clinical data. Data were extracted from the electronic medical record (EMR) system of the Affiliated Mental Health Center of Zhejiang University School of Medicine (Hangzhou Seventh People’s Hospital), a major tertiary psychiatric institution in East China. The observation period spanned 16 years, from January 1, 2010, to January 30, 2026.

The study protocol was reviewed and approved by the Ethics Committee of the hospital (Approval No. 2024-044). Given the retrospective design and complete anonymization of patient data, the requirement for individual informed consent was waived, in accordance with the Declaration of Helsinki.

### 2.2 Study population and cohort definition

The study population comprised adolescent inpatients aged 12 to 17 years at the time of their index admission.

#### 2.2.1 Inclusion criteria

1. Age: 12–17 years at the index admission.
2. Diagnosis: A primary discharge diagnosis at the index admission within the category of Mental and Behavioural Disorders (F00–F99) according to the International Classification of Diseases, 10th Revision (ICD-10).
3. Longitudinal data: At least two complete hospitalization records in the EMR system to allow trajectory analysis.
4. Follow-up duration: An interval of ≥3 years between the index admission (baseline) and the final recorded admission (endpoint), to ensure sufficient time to assess diagnostic stability. This threshold was chosen as a pragmatic balance between retaining sufficient sample size and providing enough time to observe meaningful diagnostic evolution. We recognise that this criterion focuses the cohort on patients with repeated contact and longer follow-up, which may limit generalizability to adolescents with shorter or single inpatient episodes.

#### 2.2.2 Exclusion criteria

1. A primary diagnosis of Organic Mental Disorders (F00–F09) or Intellectual Disability (F70–F79) at baseline, given their distinct etiologies compared with functional psychiatric disorders.
2. Incomplete or inconsistent diagnostic records that precluded accurate classification.

**Figure 1:**
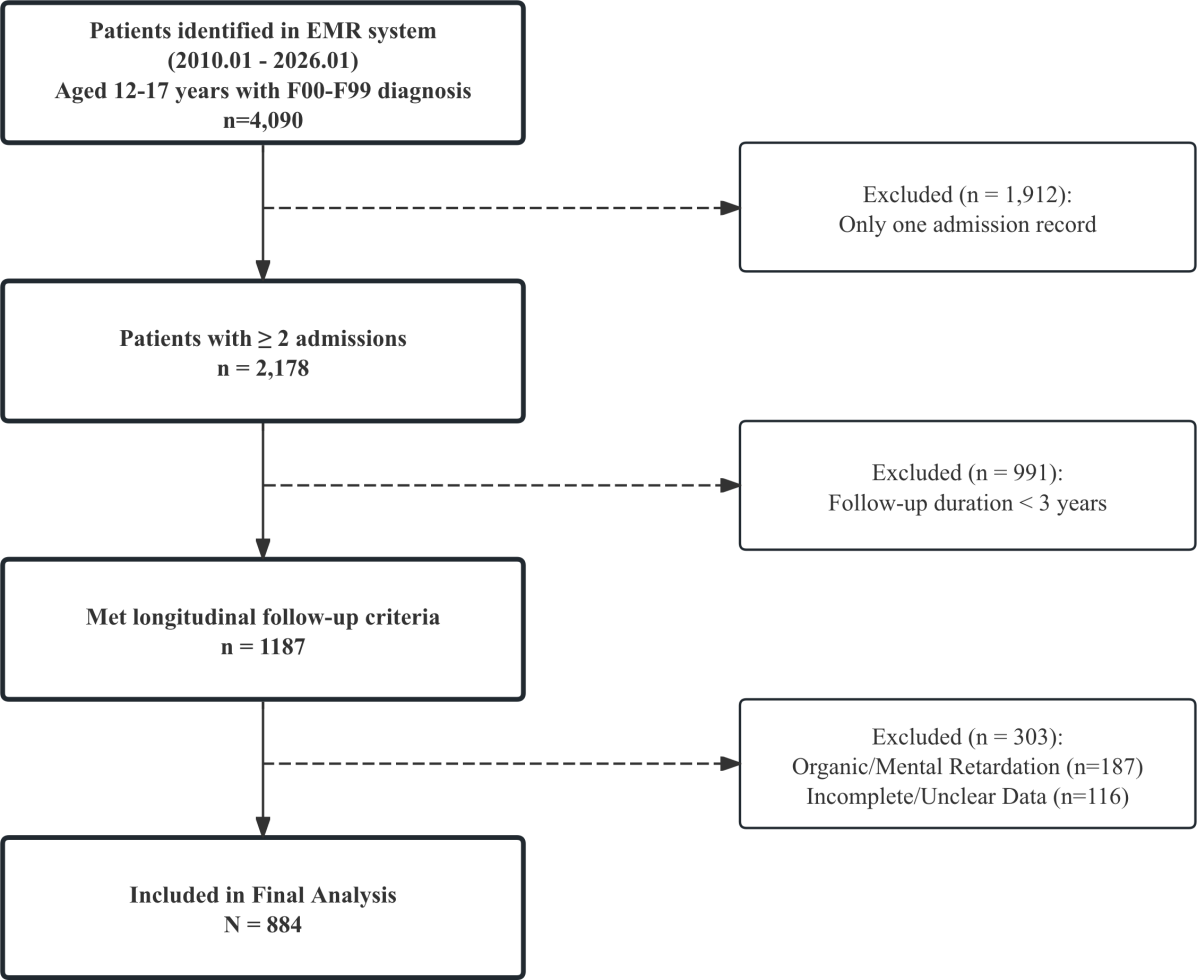
Flowchart of study participant selection.

After applying these criteria, a total of 884 adolescent patients were included in the final analytical cohort (**Figure 1** shows the flowchart of participant selection).

### 2.3 Variables and diagnostic classification

#### 2.3.1 Baseline and outcome definitions

- Baseline diagnosis: The primary discharge diagnosis recorded at the index admission.
- Final diagnosis: The primary discharge diagnosis recorded at the last hospitalization within the study window.

#### 2.3.2 Definition of severe mental illness

For the purpose of this study, severe mental illness (SMI) was operationally defined as schizophrenia spectrum disorders (F20–F29) and bipolar affective disorders (F30–F31). This definition is consistent with common clinical and public-health usage, which emphasises chronic, high-burden psychiatric conditions that typically onset in adolescence or early adulthood and are associated with substantial long-term functional impairment. All other ICD-10 primary diagnoses within F00–F99 were classified as non-SMI.

#### 2.3.3 Diagnostic grouping

To facilitate statistical analysis and visualization, individual ICD-10 codes were aggregated into clinically coherent diagnostic categories [e.g., Depressive Disorders (F32–F33), Anxiety Disorders (F40–F41), Obsessive-Compulsive Disorders (F42)]. A detailed mapping of ICD-10 codes to diagnostic categories.

### 2.4 Statistical analysis

Data management and statistical analyses were conducted using Python (version 3.9), with the pandas, scipy, lifelines, and statsmodels libraries.

#### 2.4.1 Descriptive statistics

Baseline demographic and clinical characteristics were summarised for the overall cohort and by sex. Continuous variables (e.g., age, follow-up duration) were non-normally distributed and are presented as medians with interquartile ranges (IQR). Between-group differences were assessed using the Mann–Whitney U test. Categorical variables (e.g., sex, diagnostic categories) are presented as frequencies and percentages; group comparisons were performed using the chi-squared (χ²) test.

#### 2.4.2 Diagnostic trajectory analysis

To characterise longitudinal diagnostic evolution between baseline and endpoint, we constructed a Sankey diagram visualising transitions from baseline to final diagnoses. In addition, a transition matrix was generated to calculate row-normalised conditional probabilities of diagnostic stability and conversion between specific diagnostic categories. These analyses are descriptive and intended to illustrate the main patterns of change rather than to test specific mechanistic hypotheses.

#### 2.4.3 Survival analysis

To investigate the temporal dynamics of transition to SMI, we analysed a sub-cohort of patients who were in the non-SMI group at baseline. Time zero was defined as the date of the index admission. The event of interest was the first recorded SMI diagnosis (schizophrenia spectrum disorders or bipolar disorder) during the observation period. For patients who did not develop SMI, follow-up was censored at the date of their last recorded admission.

Kaplan–Meier survival curves were used to estimate the cumulative probability of remaining SMI-free over time. The log-rank test was applied to compare survival distributions between male and female patients. The observed increase in conversion events between approximately 4 and 8 years after baseline was treated as an exploratory, descriptive finding and was not derived from a priori–defined time-to-event hypotheses.

#### 2.4.4 Risk factor analysis

To identify individual-level factors associated with conversion to SMI among patients non-SMI at baseline, we constructed a multivariable binary logistic regression model. The dependent variable was whether a patient transitioned to SMI at any point during follow-up (yes/no). Covariates included age at first admission and sex. Results are reported as odds ratios (OR) with 95% confidence intervals (CI) and visualised using a forest plot.

Given the relatively small number of events and the focus on overall risk rather than precise timing of onset, we prioritised a parsimonious logistic regression model over more complex time-to-event regression approaches (e.g., Cox models). Time to conversion is instead described via Kaplan–Meier estimates as outlined above. We did not include specific non-SMI comorbid diagnoses or intermediate diagnostic changes as predictors in this model, in order to avoid overfitting given the relatively limited number of conversion events and to maintain a parsimonious, clinically interpretable model.

All statistical tests were two-tailed, and P < 0.05 was considered statistically significant.

## 3 Results

### 3.1 Baseline characteristics of the cohort

A total of 884 adolescent inpatients met the inclusion criteria, including 547 females (61.9%) and 337 males (38.1%). Baseline demographic and clinical characteristics of the cohort are summarized in **Table 1**.

**Table 1:** Demographic and clinical characteristics of the study cohort at baseline.

Characteristic	Total (N = 884)	Male (n=337)	Female (n=547)	P-value
Age at first visit,years				<0.001
Median(IQR)	15.00[14.00,17.00]	16.00[15.00,17.00]	15.00[14.00,16.00]	
Follow-up duration,years				<0.001
Median(IQR)	4.60[3.63,6.53]	5.07[3.77,7.11]	4.29[3.56,5.98]	
Baseline SMI Status,n(%)				<0.001
SMI Group	441(49.9)	205(60.8)	236(43.1)	
Non-SMI Group	443(50.1)	132(39.2)	311(56.9)	
Primary Diagnosis,n(%)				<0.001
Schizophrenia spectrum	300(33.9)	148(45.4)	152(29.9)	
Depressive disorders	171(19.3)	37(11.0)	134(24.4)	
Bipolar disorders	141(16.0)	57(16.9)	84(15.3)	
Anxiety disorders	33(3.7)	11(3.3)	22(4.0)	
Other disorders^a^	239(27.1)	84(25.0)	155(28.3)	

Data are presented as median (IQR) for continuous variables and n (%) for categorical variables. Between-sex comparisons were performed using the Mann–Whitney U test for continuous variables and the χ² test for categorical variables.

SMI, severe mental illness.

^a^Other disorders include:childhood emotional and behavioural disorders;mood/affective disorders(unspecified);obsessive-compulsive and related disorders;dissociative disorders;disruptive behaviour/conduct disorders;sleep–wake disorders;stress-related disorders;neurodevelopmental disorders;and other unspecified mental disorders.

Several baseline characteristics differed significantly by sex. Male patients were older at first admission than females (median: 16.00 vs. 15.00 years, P < 0.001) and had a longer follow-up duration (P < 0.001).

Marked sex differences were observed in the distribution of baseline diagnoses (χ² = 53.16, P < 0.001). A higher proportion of males than females were classified as SMI at baseline (60.8% vs. 43.1%; P < 0.001). Schizophrenia spectrum disorders were the most common baseline diagnosis among males (45.4%), compared with 29.9% in females. Conversely, depressive disorders were more frequent among females (24.5%) than males (11.0%). The proportions of bipolar disorder were relatively similar between sexes (approximately 16% in both groups). Other diagnostic categories (e.g., anxiety disorders, obsessive–compulsive disorders, neurodevelopmental and behavioural disorders) were less common overall and showed sex-imbalanced distributions.

### 3.2 Longitudinal patterns of diagnostic transitions

**Figure 2:**
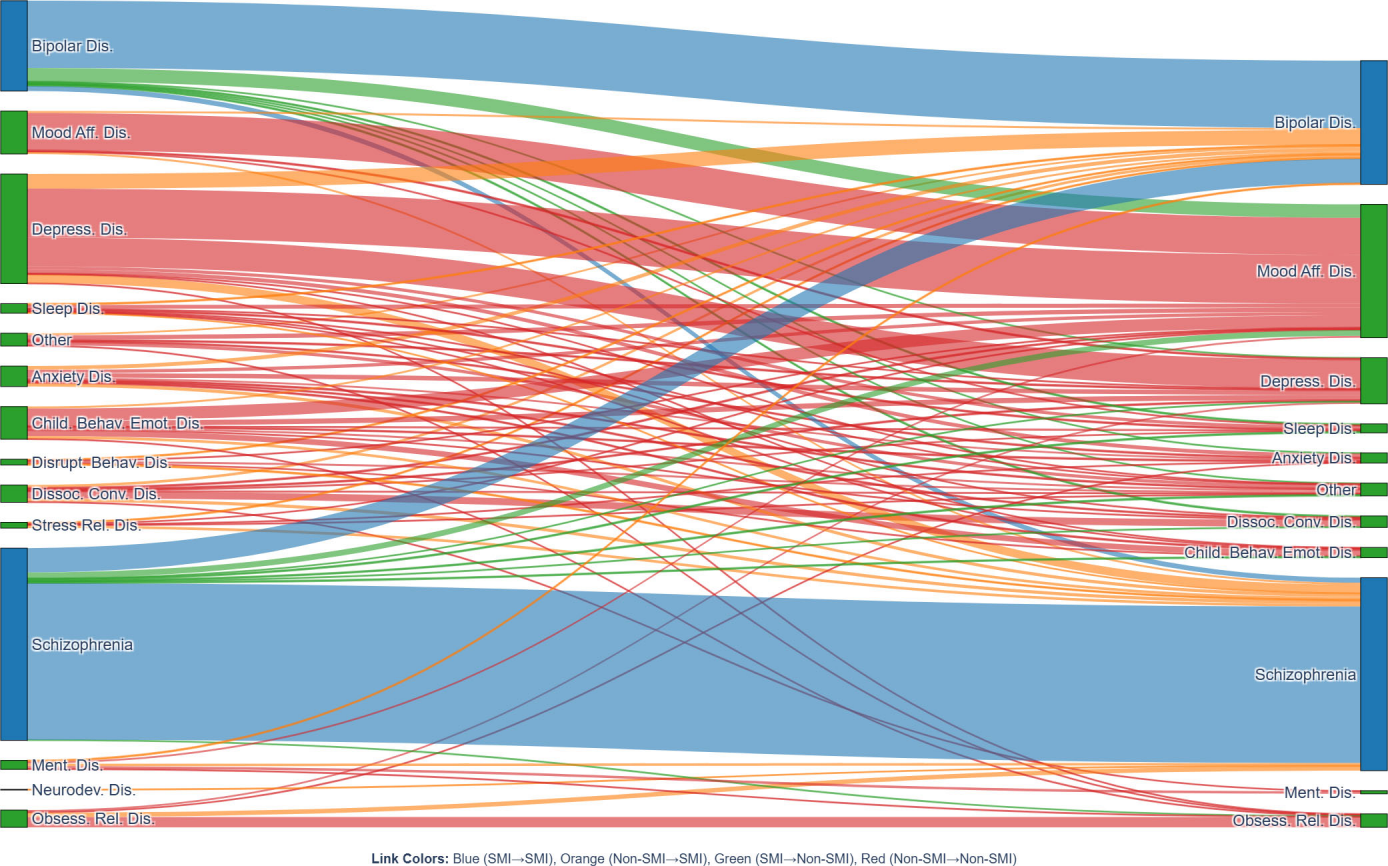
Longitudinal diagnostic transitions from baseline to final follow-up. The Sankey diagram illustrates diagnostic transitions over the 16-year study period (N = 884). The left column represents baseline diagnoses, and the right column represents final diagnoses at last follow-up. The height of each node is proportional to the number of patients in that diagnostic category, and the width of each flow corresponds to the number of patients transitioning between categories. Link colour coding: Blue: stable SMI (SMI → SMI), Orange: progression to SMI (Non-SMI → SMI), Green: reclassification from SMI to Non-SMI (SMI → Non-SMI), Red: stable Non-SMI (Non-SMI → Non-SMI). Abbreviations: SMI, severe mental illness; Schizophrenia, schizophrenia spectrum disorders; Bipolar Dis., bipolar affective disorder; Depress. Dis., depressive disorders; Anxiety Dis., anxiety disorders; Dissoc. Conv. Dis., dissociative conversion disorder; Disrupt. Behav. Dis., disruptive behaviour/conduct disorders; Child. Behav. Emot. Dis., childhood behavioural and emotional disorders; Obsess. Rel. Dis., obsessive-compulsive and related disorders; Neurodev. Dis., neurodevelopmental disorders.

**Figure 3:**
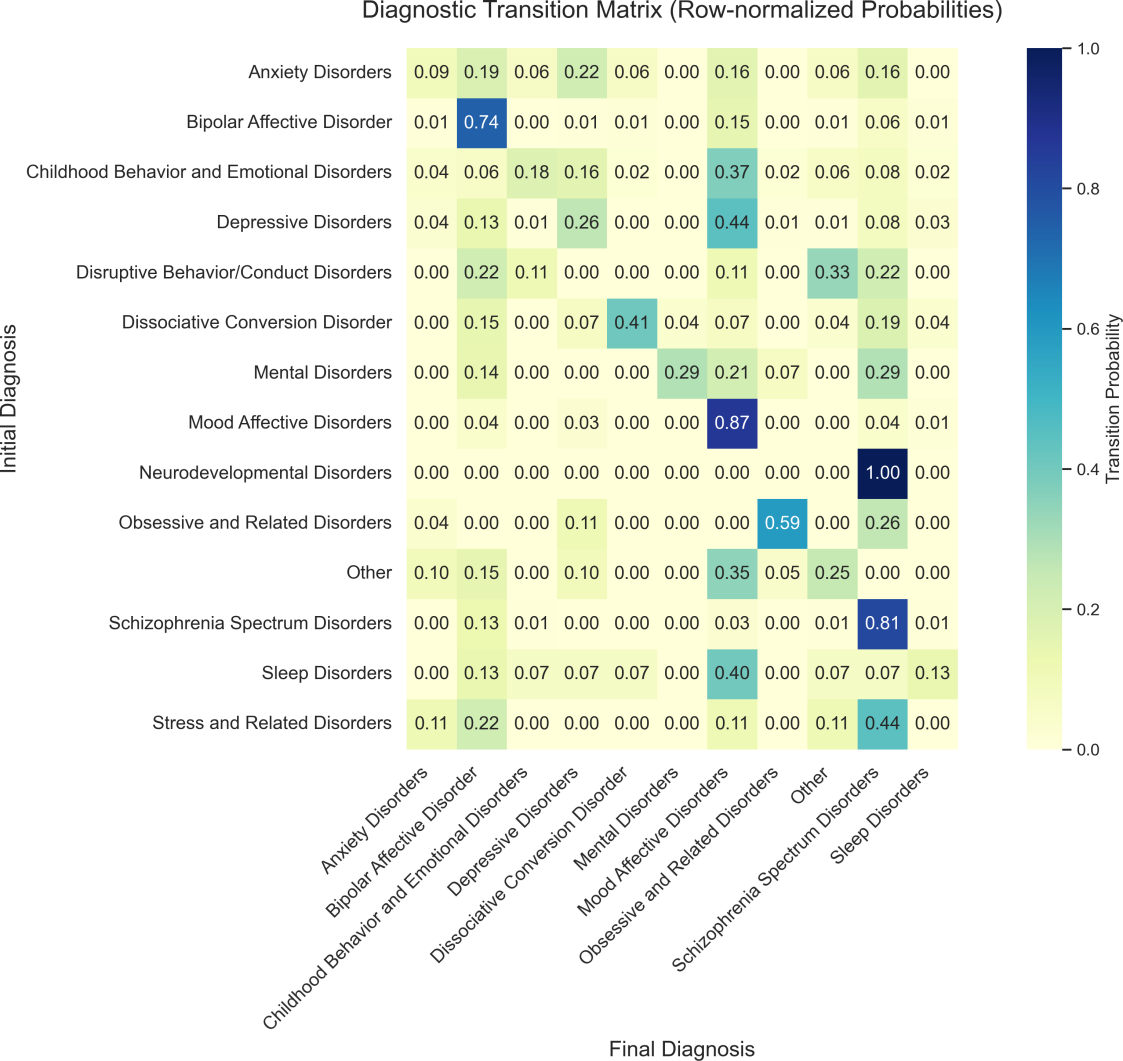
Heatmap of diagnostic transition probabilities from baseline to outcome. The matrix displays row-normalized conditional probabilities of diagnostic evolution across the follow-up period. Rows represent baseline diagnostic categories and columns represent final diagnoses at last follow-up. Each cell shows the proportion of patients with a given baseline diagnosis (row) who transitioned to a specific final diagnosis (column). Colour intensity reflects the magnitude of the probability, ranging from light yellow (low probability) to dark blue (high probability). Diagonal cells indicate diagnostic stability (same baseline and final category). Off-diagonal cells represent diagnostic conversion to a different category. Abbreviations: as in **Figure 2**.

Based on the Sankey diagram (**Figure 2**) and the transition matrix (**Figure 3**), we described diagnostic changes from baseline to the end of follow-up among the 884 adolescents. At the cohort level, SMI diagnoses showed relatively high stability, whereas non-SMI diagnoses were more diverse and displayed more frequent transitions between categories.

#### 3.2.1 Overall diagnostic stability and transitions

Across the cohort, SMI diagnoses exhibited relatively high longitudinal stability. Among patients initially diagnosed with schizophrenia spectrum disorders(n=300), 81%retained this diagnosis at final follow-up; for bipolar disorder(n=141), the corresponding proportion was 74%.

In contrast, non-SMI categories showed more heterogeneous and transitional patterns, and constituted an important upstream source of incident SMI cases. Depressive disorders were the most common non-SMI starting point for later bipolar disorder. Although 13% of patients with baseline depressive disorders(n=171) subsequently received a bipolar diagnosis, the relatively large number of adolescents with depressive disorders means that this pathway contributed a substantial proportion of new bipolar cases at the cohort level.

In addition, disruptive behaviour/conduct disorders(n=9) and stress-related disorders(n=9), despite their smaller absolute case numbers, showed comparatively high proportions of conversion to SMI. These findings suggest that, in a subset of adolescents, such presentations may be followed by more severe psychopathology. However, the absolute numbers in these subgroups were small, and these estimates should therefore be interpreted cautiously and considered exploratory.

#### 3.2.2 Sex-specific transition trajectories

**Figure 4:**
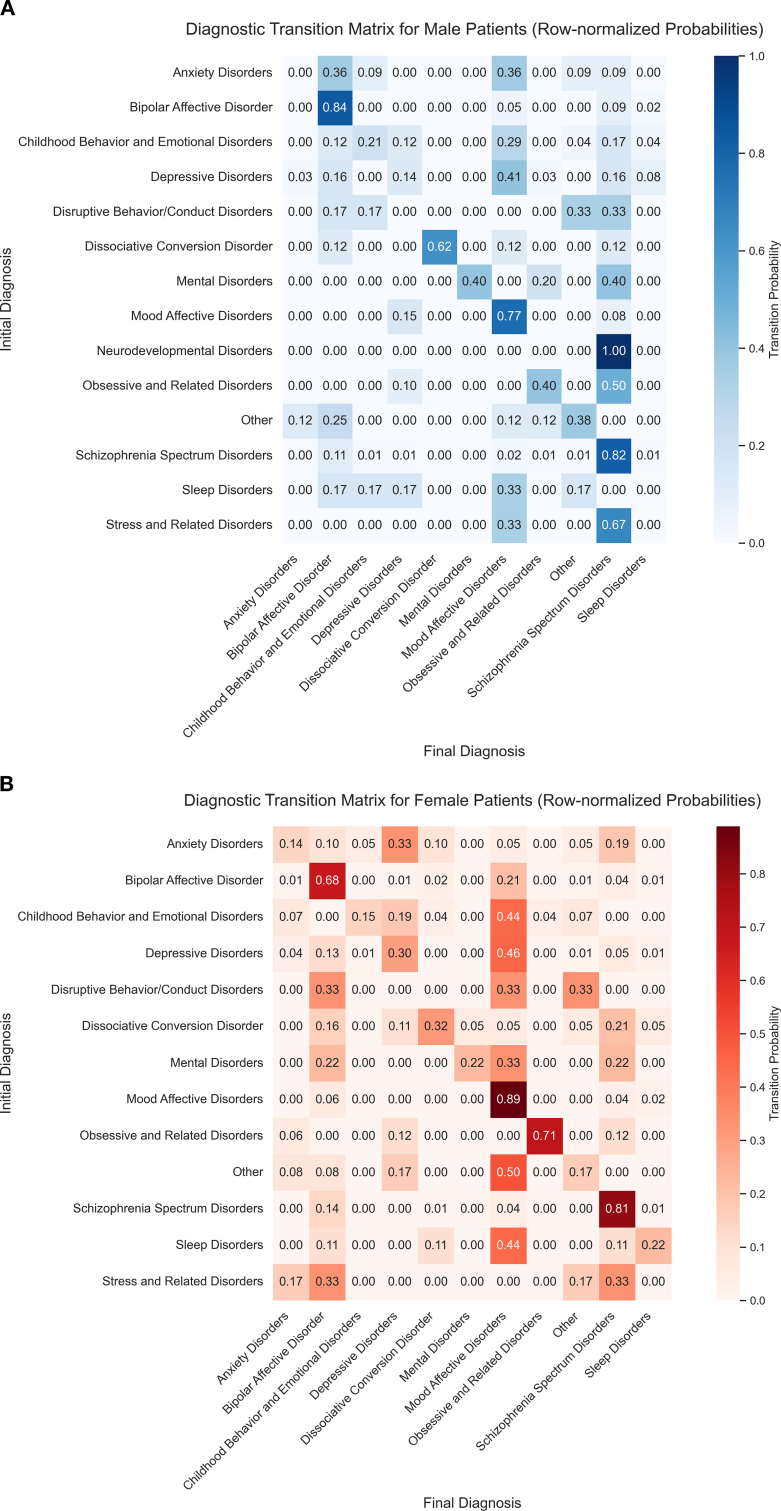
Sex-specific diagnostic transition matrices. The figure displays row-normalized diagnostic transition probabilities stratified by sex. **(A)** Male patients (n = 337): The transition matrix is visualized using a blue colour scale. It highlights relatively high transition probabilities from non-psychotic antecedents such as obsessive-compulsive and related disorders and disruptive behaviour/conduct disorders to schizophrenia spectrum disorders. **(B)** Female patients (n = 547): The transition matrix is visualized using a red–orange colour scale. It demonstrates predominant stability and transitions within the internalizing/affective spectrum (e.g., anxiety to depressive disorders), as well as elevated transition proportions from stress-related disorders to SMI. In both panels, cells show row-normalized probabilities, and colour intensity corresponds to the magnitude of the transition probability. Diagonal cells indicate diagnostic stability; off-diagonal cells indicate diagnostic conversion. Abbreviations: as in **Figure 2**.

Sex-stratified analyses (**Figure 4**) suggested some differences in diagnostic transitions between males and females. Because several diagnostic categories contained relatively few cases, these patterns should be viewed as preliminary.

##### 3.2.2.1 Male-predominant externalising and psychotic-spectrum patterns

Among males, obsessive–compulsive and related disorders(n=10) were followed by a relatively high proportion of schizophrenia spectrum diagnoses: 50% of male patients with baseline obsessive–compulsive and related disorders were ultimately classified as having schizophrenia spectrum disorders, compared with 12% of females(n=17). Disruptive behaviour/conduct disorders in males(n=6) also showed frequent transitions to SMI: 33% converted to schizophrenia spectrum disorders and 17% to bipolar disorder. In contrast, among females with disruptive behaviour/conduct disorders(n=3), transitions were split across bipolar disorder (33%), mood affective disorders (33%), and other diagnoses (33%), with 0% transitioning to schizophrenia spectrum disorders in this subgroup. Furthermore, 100% of males with neurodevelopmental disorders at baseline(n=1) were ultimately diagnosed with schizophrenia spectrum disorders.

These proportions suggest that, in some male adolescents, early presentations involving externalising behaviours or prominent obsessive–compulsive symptoms may precede later psychotic-spectrum diagnoses. Given the small numbers in these diagnostic strata, these observations require replication in larger samples before firm conclusions can be drawn.

##### 3.2.2.2 Female-predominant internalising and affective patterns

In females, diagnostic transitions tended more often to remain within internalising and affective spectra. Female patients with baseline anxiety disorders(n=21) most frequently transitioned to depressive disorders (33%), whereas male patients with anxiety disorders(n=11) showed a higher proportion transitioning to bipolar disorder (36%).

Among females with stress-related disorders(n=6), transitions were most frequently observed to bipolar disorder (33%) and to schizophrenia spectrum disorders (33%). This pattern raises the possibility that, in some female adolescents with severe stress-related presentations, these syndromes may represent early manifestations that precede different forms of SMI. Again, these estimates are based on small diagnostic subgroups and should be interpreted with caution.

### 3.3 Factors associated with transition to SMI: temporal features and risk estimates

To further examine the timing and correlates of transition from non-SMI to SMI, we performed Kaplan–Meier survival analyses and multivariable logistic regression among patients classified as non-SMI at baseline.

#### 3.3.1 Temporal course of diagnostic transition

**Figure 5:**
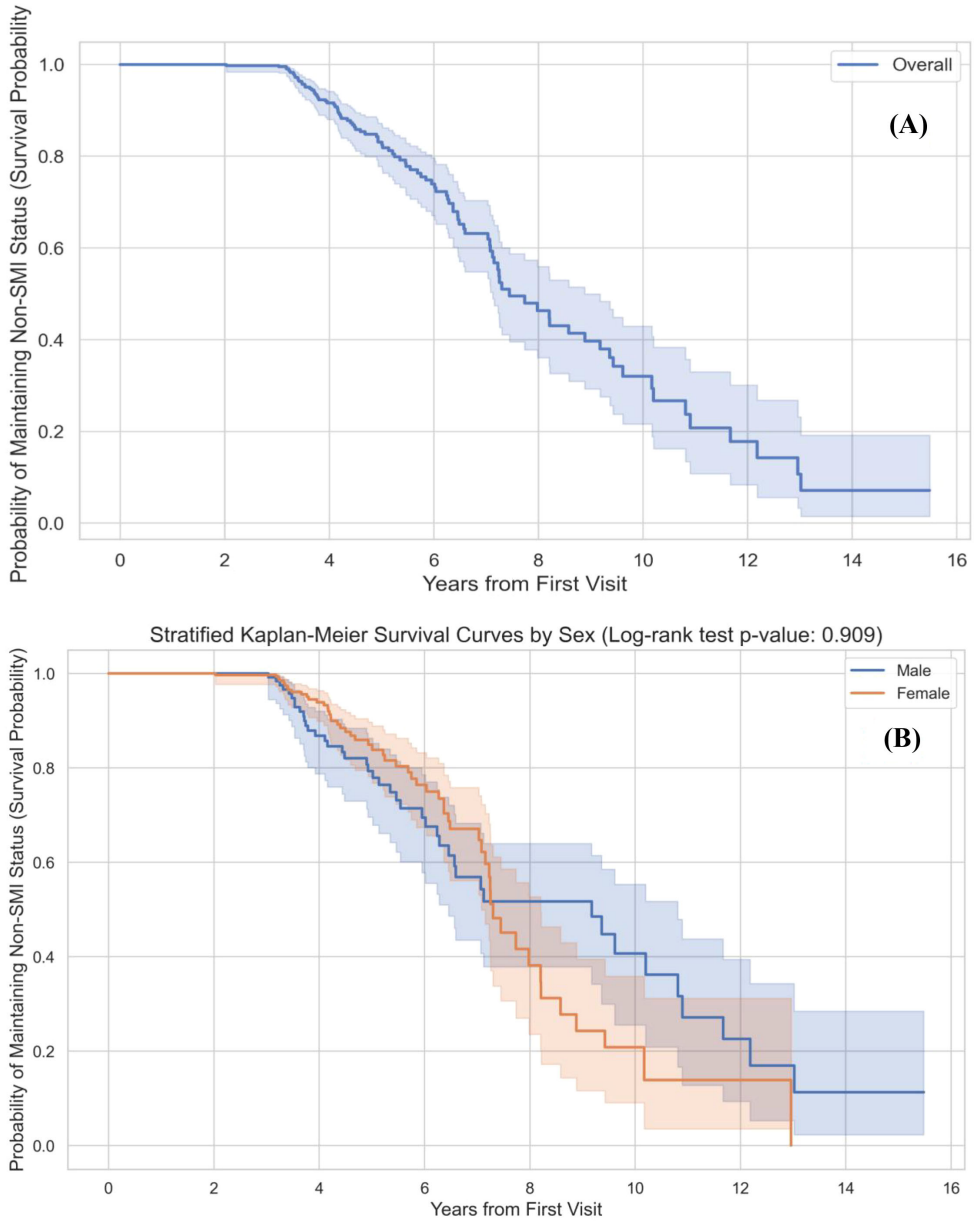
Kaplan-Meier survival analysis of time to SMI transition. The figure shows the cumulative probability of maintaining a Non-SMI diagnosis (survival) over the 16-year follow-up period, with transition to SMI as the event. **(A)** Overall cohort: The blue line represents survival for the Non-SMI sub-cohort; shaded areas indicate 95% CIs. A steeper decline between approximately years 4 and 8 indicates a period of increased diagnostic change. **(B)** Stratified by sex: Survival curves for males (blue) and females (orange). The log-rank test showed no statistically significant difference in time to SMI transition between sexes (*P* = 0.909).

Kaplan–Meier survival curves described the probability of remaining non-SMI over time among patients non-SMI at baseline (**Figure 5****).** The event was defined as the first recorded SMI diagnosis (schizophrenia spectrum disorders or bipolar disorder).

For the overall non-SMI sub-cohort (**Figure 5A**), the survival curve showed a relatively slow decline during the first 3 years, followed by a steeper decrease between approximately years 4 and 8. This pattern indicates that a sizeable proportion of diagnostic changes to SMI occurred within this period after the initial admission. However, these curves are descriptive and do not imply a discrete or sharply bounded “risk window”.

In sex-stratified analyses (**Figure 5B**), survival curves for males and females did not differ significantly (log-rank P = 0.909), suggesting broadly comparable temporal rates of transition to SMI when sex is considered alone.

#### 3.3.2 Predictors of transition to SMI

**Figure 6:**
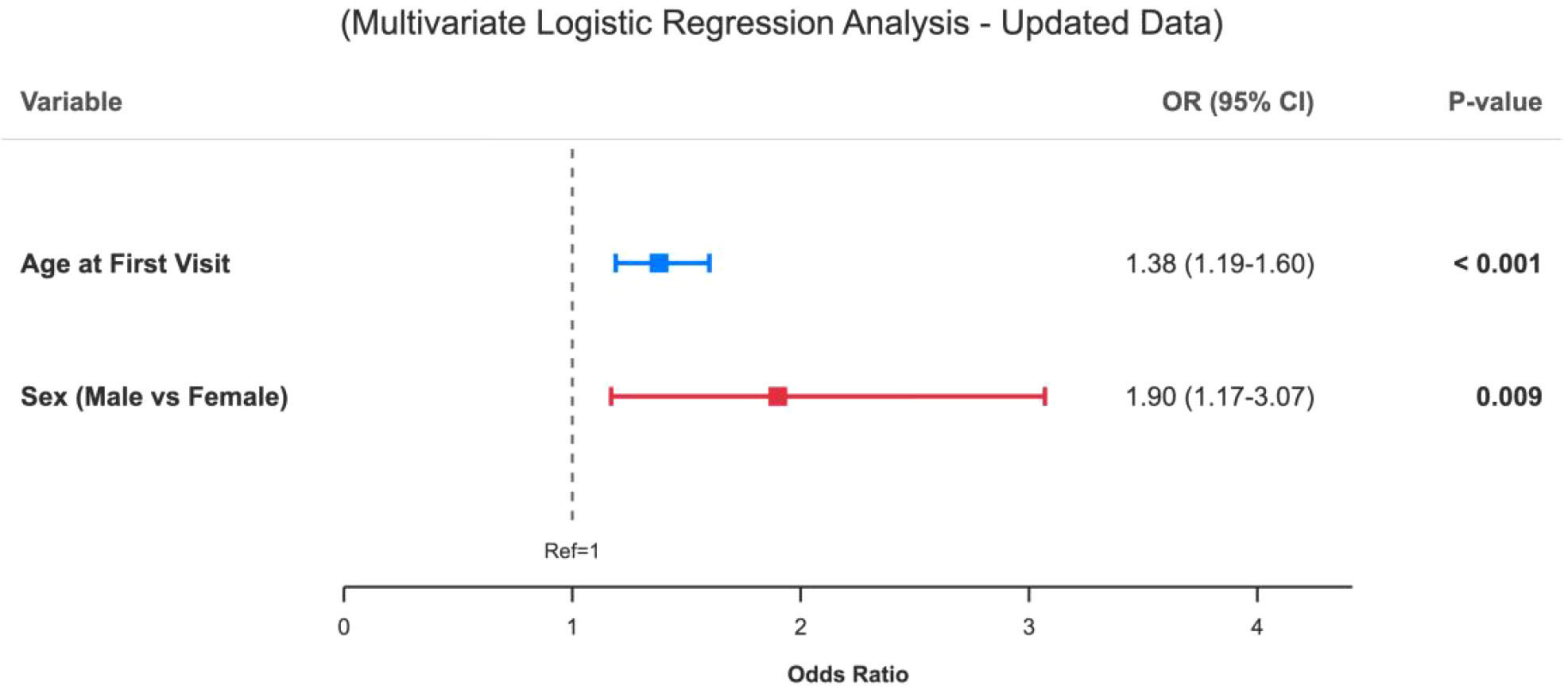
Forest plot of predictors for conversion to SMI. The forest plot depicts odds ratios (ORs) and 95% confidence intervals (CIs) from the multivariable logistic regression model for predictors of transition from Non-SMI to SMI. Age at first visit: Higher baseline age is associated with increased odds of SMI conversion (OR = 1.38, 95% CI: 1.19–1.60, P < 0.001). Sex (male vs. female): After adjustment for age, male sex is associated with higher odds of SMI conversion compared with female sex (OR = 1.90, 95% CI: 1.17–3.07, P = 0.009). The central markers represent point estimates of ORs and horizontal bars indicate 95% CIs. The vertical dashed line at OR = 1 denotes no association; values to the right indicate increased odds of SMI transition.

To identify factors associated with SMI transition, we fitted a multivariable logistic regression model among patients non-SMI at baseline (**Figure 6**).

Age at first admission was a significant correlate of later SMI diagnosis: older age at first inpatient contact was associated with increased odds of subsequent SMI (OR = 1.38, 95% CI: 1.19–1.60, P < 0.001).

Sex was also associated with transition risk after adjustment for age. Male sex was associated with higher odds of eventual SMI diagnosis compared with female sex (OR = 1.90, 95% CI: 1.17–3.07, P = 0.009).

Taken together with the survival analysis, these findings suggest that while the timing of transition (i.e., speed of change) appears similar in males and females at the group level, the overall probability of developing SMI over the follow-up period was higher in males after accounting for age at first admission. Given the observational design and the limited set of covariates, these associations should be interpreted as indicators of elevated risk rather than evidence of causal mechanisms.

## 4 Discussion

This 16-year retrospective cohort study mapped longitudinal diagnostic trajectories in 884 adolescent inpatients at a tertiary psychiatric hospital in East China. Overall, we found that severe mental illnesses (SMI)—operationally defined as schizophrenia spectrum disorders and bipolar disorder—were relatively stable once established, whereas non-SMI diagnoses showed substantial fluidity and accounted for most later SMI onsets. Within this broad pattern, we observed sex-related differences in the types of non-SMI presentations that preceded SMI and a non-uniform timing of diagnostic reclassification, with many transitions occurring several years after the index admission. Male sex and older age at first admission were associated with an increased likelihood of subsequent SMI among adolescents initially classified as non-SMI. These findings support a longitudinal, developmental perspective on adolescent diagnosis while also highlighting the limitations of cross-sectional categorical labels.

### 4.1 Overall diagnostic evolution: stability of SMI and fluidity of non-SMI

In line with previous work, schizophrenia spectrum and bipolar diagnoses in this cohort showed relatively high stability over time. Once these diagnoses were made in adolescence, most patients retained an SMI diagnosis at final follow-up. This is broadly consistent with long-term follow-up studies reporting that early-onset schizophrenia and bipolar disorder tend to remain within the psychotic or bipolar spectra, even when there are shifts between subtypes (e.g. schizophrenia vs schizoaffective disorder) (6–9, 18, 19). These observations support the idea that, when full-threshold SMI syndromes are present in youth, the core phenotype is often sustained.

By contrast, non-SMI diagnoses such as depressive, anxiety, stress-related and disruptive behaviour disorders showed much greater diagnostic variability and were the main starting point for later SMI in our sample. This pattern mirrors earlier work in child and adolescent services indicating that many non-psychotic diagnoses, particularly within internalising and externalising spectra, have modest stability and are frequently revised over repeated assessments (4, 5, 20, 21). Such “heterotypic continuity” is consistent with the concept that, in adolescence, non-specific symptom constellations can precede several different long-term outcomes rather than mapping one-to-one onto adult categories.

At the same time, diagnostic changes in our study likely reflect not only illness progression but also the accumulation of clinical information and evolving diagnostic practice. Early affective or behavioural symptoms may initially be coded under broader categories and only later reclassified as bipolar or schizophrenia spectrum disorders once mood polarity, psychotic features or functional decline become more apparent (8, 9, 14, 19). Our data therefore reinforce both the developmental plasticity of adolescent psychopathology and the inherent limitations of single time-point diagnoses.

### 4.2 Non-SMI antecedents of SMI: overall and sex-stratified patterns

Using Sankey plots and transition matrices, we described which non-SMI diagnoses most often preceded SMI in this inpatient cohort, and how these patterns differed by sex. These analyses are descriptive and, particularly for smaller diagnostic strata, should be considered exploratory.

#### 4.2.1 Depressive and other non-SMI diagnoses as upstream sources of SMI

Depressive disorders were the most frequent non-SMI starting point for later bipolar disorder in our data: 13.0% of patients with an initial depressive diagnosis were later re-diagnosed with bipolar disorder. This is broadly in keeping with international cohorts in which a minority of adolescents with major depression subsequently develop bipolar disorder (8, 13–15, 22, 23). Our findings add to this literature by providing conversion estimates in a Chinese inpatient population, although they are not intended to serve as general population risk estimates.

We also observed relatively high proportions of transitions from disruptive behaviour/conduct disorders and stress-related disorders to SMI, despite small denominators. This pattern is compatible with longitudinal evidence that severe externalising problems and trauma- or stress-related presentations in youth are associated with increased risk of later psychotic and bipolar disorders (17, 21, 24–26). However, because the absolute numbers in these subgroups were small, our estimates are imprecise and should be viewed as hypothesis-generating rather than definitive.

#### 4.2.2 Sex-related patterns in diagnostic evolution

When we stratified by sex, some differences emerged in the types of non-SMI conditions that preceded SMI. Among male adolescents, a notable proportion of those with baseline obsessive–compulsive and related disorders, neurodevelopmental disorders or disruptive behaviour/conduct disorders later received schizophrenia spectrum or bipolar diagnoses. These patterns, although based on small groups, are broadly consistent with prior studies linking childhood externalising and neurodevelopmental difficulties to later SMI, particularly in males (25–27). The observed transitions from obsessive–compulsive presentations to psychotic-spectrum diagnoses in some males also resonate with clinical reports of overlap between obsessive–compulsive and psychotic symptoms in a subset of patients (16, 28).

In females, transitions more often remained within internalising and affective spectra. Female adolescents with baseline anxiety disorders most frequently evolved towards depressive disorders, whereas male adolescents with anxiety had a higher proportion later diagnosed with bipolar disorder. This echoes broader epidemiological patterns in which early anxiety predicts unipolar depression particularly in girls, and is sometimes linked to bipolarity in both sexes (14, 29–31). We also observed that a considerable proportion of females with baseline stress-related disorders later received schizophrenia spectrum or bipolar diagnoses, suggesting that, in some cases, severe stress-related presentations may precede different forms of SMI.

Overall, these sex-stratified results tentatively suggest that, in this inpatient cohort, early externalising, neurodevelopmental and obsessive–compulsive presentations in males, and severe internalising or stress-related presentations in females, were more frequently seen among those who later developed SMI. Given the limited sample sizes in many cells and the observational nature of the data, these patterns should be interpreted with caution and require replication in larger, prospectively assessed samples before being incorporated into risk models or clinical algorithms.

### 4.3 Temporal dimension of diagnostic reclassification

Among adolescents classified as non-SMI at baseline, Kaplan–Meier analyses showed that the probability of maintaining a non-SMI diagnosis decreased most steeply between approximately 4 and 8 years after the first admission, before plateauing. This suggests that, in this cohort, diagnostic reclassification to SMI frequently occurred several years after initial inpatient contact rather than within the first 1–2 years.

This temporal pattern is broadly consistent with the age range in which incidence of schizophrenia and bipolar disorder rises most sharply—late adolescence and early adulthood (1–3, 17, 32, 33). It may also reflect the time needed for some individuals to move from non-specific or subthreshold states to full-threshold SMI, as well as changes in environmental demands (for example, transition to higher education or the workforce). However, our data capture the timing of recorded diagnostic changes, not the true onset of SMI. Symptom onset may have preceded re-diagnosis, and help-seeking, referral and admission are influenced by service availability, family resources and stigma (1, 5, 17, 34).

Therefore, the 4–8-year period we describe should not be interpreted as a sharply defined “risk window”, but rather as an empirically observed interval in which diagnostic reclassification was relatively common in this specific inpatient cohort. From a clinical standpoint, the finding supports the value of medium- to long-term monitoring of high-risk adolescents beyond short follow-up periods, but does not imply that risk is absent outside this interval.

### 4.4 Sex, age at first admission and risk of SMI

In multivariable analysis among patients non-SMI at baseline, male sex and older age at first admission were associated with higher odds of later SMI. The Kaplan–Meier curves did not show significant sex differences in the timing of transition among those who converted, implying that sex in this cohort was more related to the overall likelihood of SMI than to the speed of change.

The sex effect is consistent with epidemiological findings that males have higher incidence and earlier onset of schizophrenia spectrum disorders and are over-represented among youth who later develop psychosis after contact with mental health services (2, 6, 7, 17, 24, 33, 35). One possible explanation is that underlying risk for SMI is more likely to manifest as SMI diagnoses in males; another is that referral and admission thresholds differ, such that boys may tend to be hospitalised when symptoms are more severe or disruptive, thereby enriching the male non-SMI group for individuals closer to an SMI threshold. Our study cannot distinguish between these possibilities.

Older age at first admission was also associated with later SMI. This could reflect a longer duration of untreated illness before first hospitalisation, proximity to the peak age of onset of schizophrenia and bipolar disorder, or more stringent admission criteria for older adolescents (1–3, 7, 17, 32, 33, 36). Because we lacked systematic data on duration of untreated illness, premorbid functioning, neurocognition, family history and environmental exposures, these mechanisms remain speculative. Prospective studies with richer clinical and biological characterisation are needed to clarify how sex and age at first contact contribute to SMI risk trajectories (25, 26, 32, 33, 37).

### 4.5 Strengths and limitations

This study has several strengths. It draws on a 16-year EMR-based cohort from a large tertiary psychiatric hospital, providing rare long-term data on diagnostic stability and change in Chinese adolescent inpatients, a group under-represented in the literature. The combination of Sankey diagrams, transition matrices, survival analysis and multivariable regression enabled us to examine diagnostic trajectories descriptively and inferentially, including sex-stratified and temporal patterns. Our operational definition of SMI as schizophrenia spectrum disorders and bipolar disorder aligns with common usage in service planning and much of the psychosis and bipolar risk literature, and was chosen because these conditions share clinical severity, functional impact and treatment needs (3, 8, 14, 32, 37, 38).

Several limitations must also be acknowledged. First, the retrospective design based on routine EMR data makes the findings susceptible to information and classification bias. Diagnoses were made by treating clinicians without structured research interviews and may have been influenced by evolving diagnostic systems, clinical experience and service pressures over time (4, 5, 34, 39). In addition, some diagnostic categories that are relatively rare in adolescent inpatient settings in our context—such as eating disorders, personality disorders and substance use disorders—were either infrequent or not represented as primary discharge diagnoses in this cohort, and were therefore not analysed as separate groups. This further limits the generalisability of our findings to these conditions. We lacked consistent measures of symptom severity, functioning and family history; consequently, we could not examine staging or outcomes in detail.

Second, the sample consists of inpatients at a tertiary centre and likely represents a risk-enriched group with more severe and complex presentations than typical community or outpatient populations. Conversion proportions, particularly in small subgroups, should therefore be interpreted as applying to a highly selected clinical population, not to the general adolescent population (17, 34, 38).

Third, some specific transition pathways—especially those originating from neurodevelopmental disorders, disruptive behaviour/conduct disorders and obsessive–compulsive and related disorders—were based on small numbers, leading to unstable estimates and wide confidence intervals. Although these patterns are broadly consistent with prior literature, we consider them exploratory and in need of replication (25–27).

Fourth, our primary analyses focused on first and last recorded diagnoses within the observation window. Intermediate diagnostic changes, comorbidity, and treatment exposures were not systematically modelled, yet these factors may be important for understanding staging, remission and relapse (19). Finally, residual confounding by unmeasured variables—such as socio-economic adversity, trauma, substance use and cognitive functioning—cannot be ruled out. Prospective, multi-site studies with standardised diagnostic interviews, structured staging assessments and multimodal biomarkers will be essential to validate and refine our observations (32, 37, 40).

### 4.6 Clinical and research implications

Taken together, our findings support viewing adolescent psychiatric diagnosis as a dynamic process that unfolds over several years. SMI diagnoses were relatively stable once assigned, but a substantial minority of adolescents with non-SMI diagnoses eventually received SMI diagnoses, often after a considerable delay. In this risk-enriched inpatient cohort, non-SMI presentations linked to later SMI showed some sex-related differences, and most diagnostic reclassifications from non-SMI to SMI occurred between roughly 4 and 8 years after first admission. Male sex and older age at first admission were associated with higher odds of later SMI (41).

Clinically, these results argue against treating the first inpatient diagnosis in adolescence as definitive, particularly in those with severe or atypical non-SMI presentations. For adolescents requiring hospitalisation with prominent externalising, neurodevelopmental or obsessive–compulsive symptoms (more frequent among males in our data), and those with severe internalising or stress-related presentations (more frequent among females), it may be prudent to adopt a longitudinal, staging-informed approach that keeps the possibility of later SMI in view. Medium- to long-term follow-up beyond the short time frames used in many early intervention programmes may be necessary to detect later-emerging SMI and to adapt treatment over time.

From a research perspective, our study underscores the need for prospective, multi-centre cohorts in diverse cultural and service contexts, integrating dimensional psychopathology, neurocognition, biological markers and environmental risks. Such work could clarify which combinations of early symptoms, sex, age at onset and contextual factors are most informative for risk stratification, and how these can be translated into feasible monitoring and intervention strategies in routine adolescent mental health care (42).

## 5 Conclusion

This 16-year EMR-based cohort study indicates that, in adolescent inpatients, schizophrenia spectrum and bipolar diagnoses are relatively stable over time, whereas non-SMI diagnoses show greater fluidity and constitute an important source of later SMI. In this risk-enriched clinical population, certain non-SMI presentations—such as depressive, externalising, neurodevelopmental, obsessive–compulsive, anxiety and stress-related disorders—more frequently preceded SMI, with some sex-related differences in these patterns. Diagnostic reclassification from non-SMI to SMI often occurred several years after first admission, and male sex and older age at first admission were associated with a higher likelihood of subsequent SMI.

These findings suggest that adolescent inpatient diagnoses, particularly non-SMI diagnoses, should be viewed as provisional within a longer developmental course. For adolescents with severe or atypical non-SMI presentations, it may be appropriate to ensure medium- to long-term clinical follow-up and to remain alert to the possible emergence of SMI, while recognising that only a minority will convert. Future prospective studies in larger and more diverse samples are needed to refine these preliminary risk indicators and to develop practical, developmentally informed tools for early identification and monitoring of adolescents at elevated risk for SMI.

## Data Availability

The raw data supporting the conclusions of this article will be made available by the authors, without undue reservation.
